# Crude Oil Treatment Leads to Shift of Bacterial Communities in Soils from the Deep Active Layer and Upper Permafrost along the China-Russia Crude Oil Pipeline Route

**DOI:** 10.1371/journal.pone.0096552

**Published:** 2014-05-02

**Authors:** Sizhong Yang, Xi Wen, Liang Zhao, Yulan Shi, Huijun Jin

**Affiliations:** 1 State Key Laboratory of Frozen Soils Engineering (SKLFSE), Cold and Arid Regions Environmental and Engineering Research Institute (CAREERI), Chinese Academy of Sciences, Lanzhou, Gansu, China; 2 College of Electrical Engineering, Northwest University for Nationalities, Lanzhou, Gansu, China; University of Kansas, United States of America

## Abstract

The buried China-Russia Crude Oil Pipeline (CRCOP) across the permafrost-associated cold ecosystem in northeastern China carries a risk of contamination to the deep active layers and upper permafrost in case of accidental rupture of the embedded pipeline or migration of oil spills. As many soil microbes are capable of degrading petroleum, knowledge about the intrinsic degraders and the microbial dynamics in the deep subsurface could extend our understanding of the application of *in-situ* bioremediation. In this study, an experiment was conducted to investigate the bacterial communities in response to simulated contamination to deep soil samples by using 454 pyrosequencing amplicons. The result showed that bacterial diversity was reduced after 8-weeks contamination. A shift in bacterial community composition was apparent in crude oil-amended soils with *Proteobacteria* (esp. *α-*subdivision) being the dominant phylum, together with *Actinobacteria* and *Firmicutes*. The contamination led to enrichment of indigenous bacterial taxa like *Novosphingobium*, *Sphingobium*, *Caulobacter*, *Phenylobacterium*, *Alicylobacillus* and *Arthrobacter*, which are generally capable of degrading polycyclic aromatic hydrocarbons (PAHs). The community shift highlighted the resilience of PAH degraders and their potential for *in-situ* degradation of crude oil under favorable conditions in the deep soils.

## Introduction

Increasing human activities related to petroleum extraction and transport in cold regions can result in the release of crude oil and petroleum products in the environment, and have severe ecological and socioeconomic consequences. Thus, pipeline leakage has been reported to be the main contamination source [1]. For example, over 100,000 tons of crude oil was released to Usinsk (65°N) in the Kolva River Basin from pipeline systems, leading to a massive local biodiversity loss as well as some other long-term environmental impacts [1], [2]. Moreover, relatively small amount of oil released by pipeline systems occurs frequently in cold regions, and are generally invisible or difficult to measure [3]. Thus, these contaminants will be present in the cold environment and be subject to *in-situ* degradation by the indigenous microorganisms [4].

Spilled oil can migrate in the active layer when it is unfrozen or thawing. During freeze-thaw cycles, hydrocarbons can migrate ahead of the freezing front and accumulate in the permafrost interface [5]. Even the permafrost is not an impermeable barrier. Thus, hydrocarbons can move through active layer into frozen soil via cracks or fissures or unfrozen pore water [6], [7] and oil components were even observed to penetrate into completely ice-saturated soils [5]. Hydrocarbons which are limited to the bottom of active layer can move further downward if the top part of permafrost is thawing. Therefore, this migration is more likely to happen in thermally unstable permafrost area due to climate warming. However, minimal attention has been given to the attenuation of petroleum spillage in permafrost table, owing to the belief that the permafrost is an impermeable barrier, which constrains contaminant transport downward [8].

Wherever petroleum is found in freezing and frozen soils, they can be degraded by hydrocarbon-degrading microorganisms [9]–[11]. Cold-adapted intrinsic bacteria can be still active in cold regions and have potential to *in-situ* break down petroleum pollutants, even though they are influenced by environmental limitation [11]. Most research has considered hydrocarbon degradation in the active layer, while a substantial number of hydrocarbon degraders have also been detected in permafrost soils [12]. These degraders could use hydrocarbon contaminants which migrate downward in the deep subsurface soils, sustain and enhance number and proportion of hydrocarbon-degrading microbes [4], [12]–[14].

Recently, the China-Russia Crude Oil Pipeline (CRCOP) was built in the permafrost regions in northeastern China. This buried pipeline goes through permafrost-affected forests, wetlands and distinct cold ecosystems in northeastern China [15]. The deep active layer and upper permafrost may be contaminated either directly by accidental leakages from the pipeline or indirectly by migrated hydrocarbons. For another, permafrost there is warm, thin and sensitive to climatic warming [16]. The deepening of the seasonal active layer can provoke changes in microbial processes. Microorganisms of the deep subsurface could be potentially activated and involved in onsite bioremediation.

Nevertheless, the comprehension of the diversity and dynamics intrinsic cold-adapted degraders are indispensable to develop bioremediation strategies. Yet, few studies have addressed the microbial communities particularly hydrocarbon degraders inhabiting in the deep subsurface along the CRCOP pipeline. In the present study, deep soils samples from four high-risk permafrost sections along the pipeline were subjected to identical contamination with crude oil. The purpose is to understand community shifts over time and to identify potential microorganisms capable of hydrocarbon degradation in the deep active layer and upper permafrost. In order to accomplish this goal, bacterial communities were characterized using multiplexed 454 pyrosequencing of 16S rRNA gene amplicons.

## Materials and Methods

### Sample Collection

Soil samples were collected at the bottom active layer and upper permafrost from four different plots in Walagan North (WN, 52°43′ N, 124°30′ E), Walagan (WL, 52°26′ N, 124°40′ E), Tayuan (TY, 51°27′ N, 124°15′ E) and Jiagedaqi (JQ, 50°41′ N, 124°17′ E) (Table S1). These areas are at high risk of oil spills (referring details to [16]). The soil samples were collected in the state-owned land which is open for scientific research. No specified permissions are required for these sampling sites, which are not natural reserve and did not involve endangered or protected species.

### Contamination Experiment

After removing visible gravels, fresh soil material (10 g) from different soil layers were weighed into 50 ml glass jars and closed with a screw cap containing a septum. The glass jar was fitted with U-shaped (Gooseneck) tube. The neck of U-shaped glass tube was filled with a sterile filter in order to let fresh air in. Soils were slightly crushed with sterilized mortar and pestle in a clean bench. After that, the crude oil from the CRCOP pipeline was added into soils at a percentage of 30% (volume/weight) and well mixed with stirring rod. Each layer had three replicates. Incubation of contaminated soils was set at 25°C. DNA was directly extracted from soil samples 8 weeks after the treatment.

### DNA Extraction, PCR and Pyrosequencing

For each sample, triplicate DNA aliquots were extracted with Power Soil DNA Isolation Kit (MOBIO, USA) according to the manufacturer’s instructions. Bacterial primer set of 8F (5′-3′ GAGTTTGATCCTGGCTCAG) and 533R (5′-3′ TTACCGCGGCTGCTGGCAC) was used to amplify the V1–V3 regions of 16S rDNA. Barcodes were incorporated at the 5′ end of the forward primer to allow multiplexing pyrosequencing. The PCRs were carried out in triplicate 20 µL reaction volumes containing 0.5 µL DNA template, 250 µM dNTPs, 0.1 µM of each primer and 2.5 U FastPfu Polymerase (Applied Biosystems) and appropriate 5× FastPfu buffer. The PCR amplification was conducted under the following conditions: initial denaturation at 95°C for 2 min; 25 cycles at 94°C for 30 s, 55°C for 30 s, and 72°C for 30 s, and a final extension at 72°C for 5 min. PCR amplicon libraries were prepared by combining three independent PCR products for each sample to minimize the impact of potential early round PCR errors. After purification with MiniElute PCR purification kit (Qiagen, Germany), the PCR products were quantified using the GeneQuant pro system (GE Healthcare) and then mixed accordingly to achieve the equal concentration in the final mixture. Then the equalized PCR products were submitted for pyrosequencing on a Roche GS-FLX Titanium platform at Majorbio Bio-pharm Technology (Shanghai, China).

### Data Analysis

Sequence analysis was carried out using mothur software platform [17]. First, barcodes and PCR primer were trimmed and sequences with ambiguous bases, or with more than eight homopolymer nucleotides were removed. Then, unique sequences were aligned to the reference SILVA database by default settings. After chimera was checked, bad sequences were further removed. Sequences passing these screens were then used to produce distance matrix. The OTUs (operational taxonomic units) were classified at similarities of 97% (species) and 95% (genus). Predictive rarefaction curve and the richness indices (Shannon diversities and the Chao1 richness) were generated by mothur and *R* packages [18]. A heatmap across all samples was generated by *R* packages of pheatmap v. 0.7.4 [19]. The plots of dominant phylotypes were produced by *R* package of ggplot2 v. 0.9.3.1 [20]. The ordination diagrams were generated by *R* package of vegan v.2.0–7 [21]. The clustering within the dendrogram of the clean and oily samples was statistically tested by unifrac.weighted command of mothur.

### Data Availability

Sequence data of indigenous bacterial communities has been deposited at NCBI Sequence Read Archive under accession number SRR548601, and pyresequencing data of crude oil treated bacteria in this experiment can be accessed in the subset of SRR1180575. Both data can be accessed under the study accession SRP015314.

## Results

**Figure 1:**
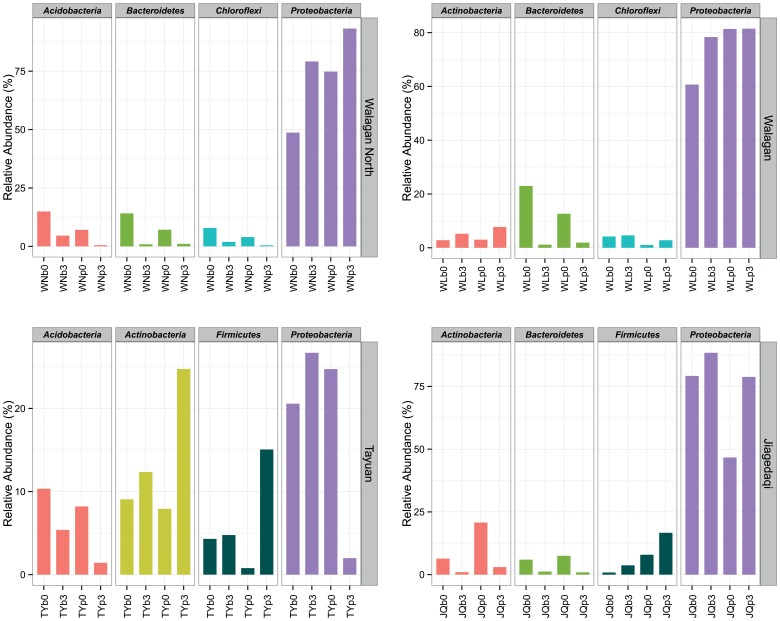
Plot showing the top four bacterial taxa in each sample based on the mean value of relative OTU abundance in pristine and contaminated communities. For the sample labels, the first two uppercase letters are site names abbreviation, followed by lowercase alphabet “b” and “p” specified the bottom active layer and upper permafrost, and last numeric suffix “0” or “3” indicates innate soils (without pollutants) and 30% (v/w) treatment, respectively.

Briefly, contamination for 8 weeks resulted in generally decreased total number of OTUs compared with the respective innate samples (Fig. S1 and S2). One of the major changes is the increase of the relative abundance of several dominant bacterial phyla like the *Proteobacteria* (mainly alpha-subdivision), *Actinobacteria* and *Firmicutes* (Fig. 1). Among them, *Proteobacteria* particularly increases abundance in most samples except TYp3, with peak value of 92.61% in WNp3 sample. The enrichment of *Actinobacteria* mainly occurs in Tayuan samples, occupying abundance up to 24.74% in TYp3. In addition, *Bacteroidetes*, *Firmicutes*, and *Chloroflexi* are also relatively abundant, but with large variation between samples. Members from *Firmicutes* show noticeable increase in samples of WNb3 (11.17%), TYp3 (15.06%) and JQp3 (17.86%). *Chloroflexi* shows decrease in Walgan samples while slight increases in WN sample, with an average abundance lower than 5% across the bacterial assemblages. On the other hand, the relative abundance of *Bacteroidetes* and *Acidobacteria* declines in oil-amended soils.

**Figure 2:**
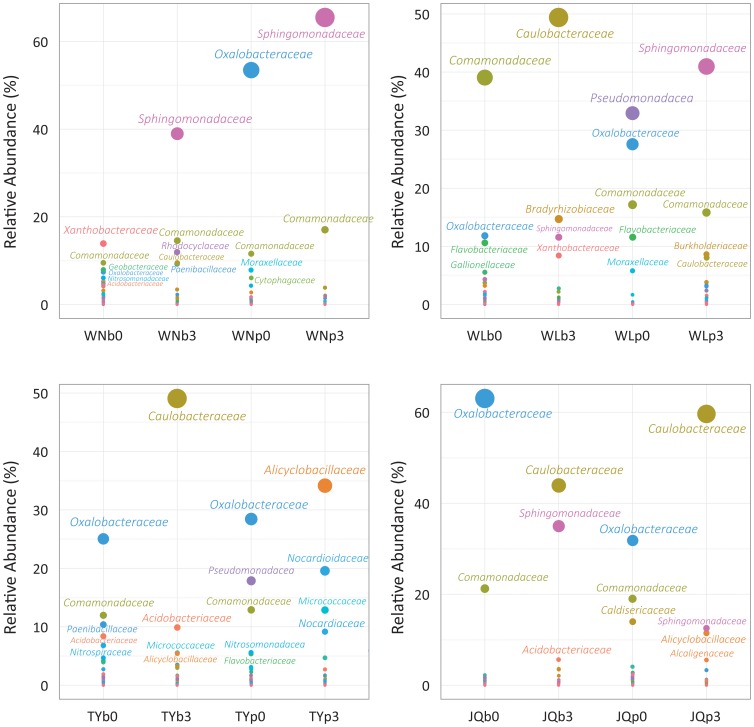
The dominant families in the pristine and oily bacterial communities in each sample. The dot size is proportion to the relative abundance of each family. Family names with average abundance higher than 5% were showed.

At rank family (Fig. 2), *Sphingomonadaceae* became the most dominant group in oil-amended Walagan North (WN) samples, occupying 38.81% in WNb3 and 65.22% in WNp3. In addition, *Comamonadaecae* were also abundant in polluted deep active layer (WNb3, 14.50%) and permafrost samples (WNp3, 16.98%). The structure is unlike the innate communities which were dominated by *Xanthobacteraceae* in the deep active layer (WNb0, 13.64%) and *Oxalobacteraceae* (WNp0, 53.36%) in permafrost, respectively.

In the oiled Walagan soils (WL), *Caulobacteraceae* (48.78%) and *Sphingomonadaceae* (40.67%) significantly dominated deep active layer and permafrost, respectively; while *Comanonadaceae* (WLb0, 38.87%), *Pseudomonadaceae* (WLp0, 32.89%) and *Oxalobacteraceae* (WLp0, 27.56%) prevail the respective indigenous communities.

Tayuan soils after treatment were mainly composed of *Caulobacteraceae* (48.89%) and *Acidobacteriaceae* (9.83%) in the deep active layer, while the innate communities (TYb0) were dominated by *Oxalobacteraceae* (24.89%) and *Comamonadaceae* (11.88%). For upper permafrost samples, *Alicyclobacillaceae* (33.99%) and *Nocardioidaceae* (19.47%) were very common in oiled samples (TYp3), in contrast to the prevalence of *Oxalobacteraceae* (28.39%) and *Pseudomonadaceae* (17.86%) in clean soil (TYp0).

Among Jiagedaqi samples, significant increase of *Caulobacteraceae* could be observed in both the JQb3 (42.61%) and JQp3 (58.53%) bacterial profiles. Besides, *Sphingomonadaceae* were considerably enriched to 33.94% and 12.32% in the corresponding layers. Moreover, *Alicyclobacillaceae* became the third group in sample of permafrost (JQp3, 11.27%). These features differentiate the oily samples from the pristine ones which were mainly composed of *Oxalobacteraceae* (JQb0∶63.05%, JQp0∶31.20%), *Comanonadaceae* (JQb0∶21.24%, JQp0∶18.63) and *Caldisericaceae* (JQp0∶13.69%).

**Figure 3:**
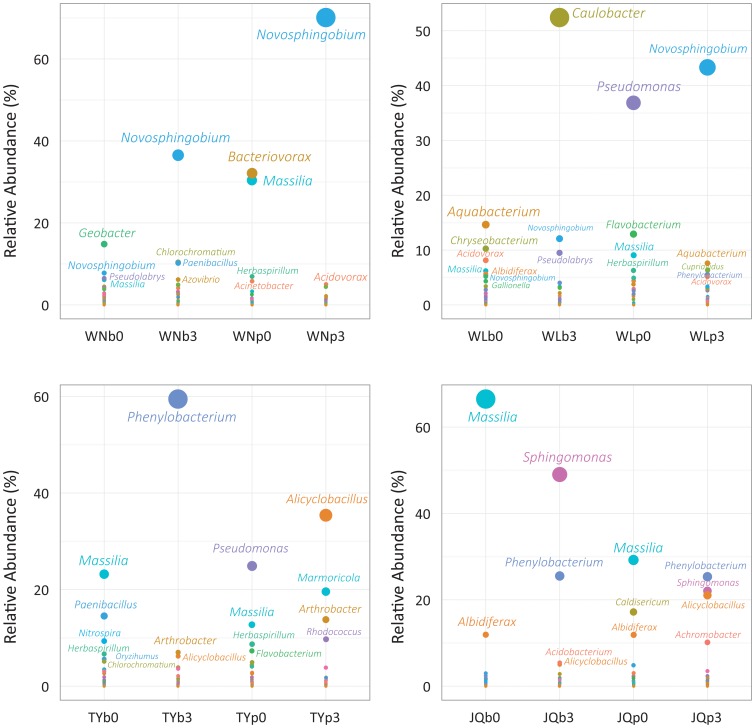
The dominant genera in the clean and oiled bacterial communities in each sample. The size of dots is corresponding to the abundance of each bacterial genus. Names of the genera with average abundance greater than 5% were only showed.

Changes in communities could be also observed at genus level (Fig. 3). For the pristine WN soils, the common genera are *Geobacter* (14.83%) in the active layer (WNb0) and *Bacteriovorax* (32.13%), *Massilia* (30.42%) in the permafrost sample (WNp0). After treatment, *Novosphingobium* is the most dominant genus in deep active layer (WNb3, 36.53%) and particularly in permafrost sample (WNp3, 70.09%). The second groups are *Chlorochromatium* (10.38%, WNb3) for active layer and *Acidovorax* (5.0%, WNp3) for permafrost samples, respectively. Taking account of the branch length from the dendrogram and the weighting, the pristine and oiled WN samples have a significantly different structure for each layer (WNb3-WNb0, *p*<0.001; WNp3-WNp0, *p*<0.001).

Among Walagan samples, the oil-amended communities were characterized by abundant *Caulobacter* (52.41%) and *Novosphingobium* (12.08%) in the deep active layer (WLb3), and *Novosphingobium* (43.31%) and *Aquabacterium* (7.59%) in the permafrost sample (WLp3). In contrast, the *Aquabacterium* (14.63%) and *Pseudomonas* (23.68%) which dominated the innate communities in deep active layer (WLb0), *Pseudomonas* (36.85%) and *Flavobacterium* (12.90%) in permafrost (WLp0), respectively. The statistic indicated that the clustering is clearly different between the pristine and oiled samples (WLp3-WLp0, *p*<0.001; WLb3-WLb0, *p*<0.001).

In Tayuan samples, crude oil resulted in substantial increase of *Phenylobacterium* (59.47%), *Arthrobacter* (6.98%) and *Alicyclobacillus* (6.23%) in deep active layers sample (TYb3), and *Alicyclobacillus* (35.39%), *Marmoricola* (19.58%) and *Arthrobacter* (13.79%) in permafrost sample (TYp3). In the clean communities, *Massilia* (23.17%) and *Paenibacillus* (14.52%) prevail in soil of deep active layer (TYb_0_), while *Pseudomonas* (24.87%) and *Massilia* (12.70%) dominated in the upper permafrost soil (TYp0). However, the clustering within the tree is not statistically significant for both layer (TYp3-TYp0, *p* = 1; TYb3-TYb0, *p* = 0.528).

For Jiagedaqi samples, the genera of *Phenylobacterium* (JQb3∶25.52%; JQp3∶25.33), *Sphingomonas* (JQb3∶49.01%; JQp3∶22.09%), *Alicyclobacillus* (JQb3∶5.01%; JQp3∶21.06%) and *Achromobacter* (JQp3∶10.17%) prevailed in oily samples, while the pristine samples contains abundant *Massilia* (JQb0∶66.53%; JQp0∶29.22%), *Albidiferax* (JQb0∶11.93%; JQp0∶11.87%) and *Caldisericum* (JQp0∶17.16%). According to the statistic, it is significantly different between the clean and oily samples for both layers (JQp3-JQp0, *p*<0.001; JQb3-JQb0, *p*<0.036).

**Figure 4:**
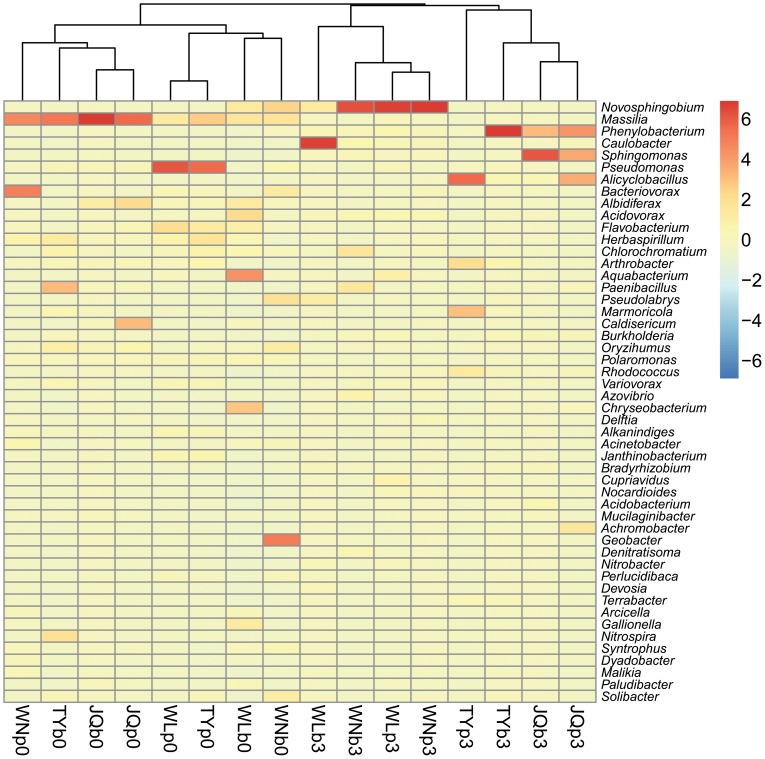
Pheatmap showing the top 50 genera detected in the baterial libraries based on the bray-curtis distance.

The hierarchical heatmap was generated in light of the top 100 abundant bacterial genera because there are so many OTUs across the clean and oily samples (Fig. 4). Briefly three clusters were suggested. The cluster in the middle is primarily composed of the pristine (“clean”) samples from deep active layer (b0) and upper permafrost (p0). The other two groups mainly constitute samples of the north and south parts along the pipeline, respectively. One of them is totally made up of the treated samples of TY and JQ from the south part, the other was composed by WN and WL but with two exceptions of WLb0 and WNb0. Moreover, the color of each cell in heatmap also displayed the prevalent group in different samples, *e.g*., *Massilia* and *Pseudomonas* in the innate bacterial communities, and *Novosphingobium* in the oil-amended WL and WN samples. Furthermore, the prevalent lineages in the southern samples (TY and JQ) are classified to the genera of *Sphingomonas*, *Phenylobacterium* and *Alicyclobacillus*. Some other less abundant groups also show slight, asymmetric or irregular shifts in abundance. In addition, the communities after treatment generally clustered closely to the same site.

**Figure 5:**
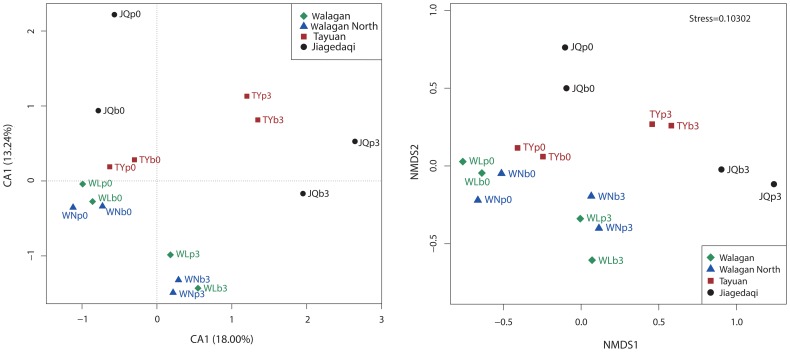
Correspondence analysis (CA) (left) and non-metric multidimensional scaling (NMDS) ordination (right) for bacterial communities of both innate and contaminated samples. Note: The configuration of NMDs was rotated to have the greatest variation along the first axis.

The CA ordination showed that the indigenous bacterial profiles either from the deep active layer or permafrost mapped together in the upper left of the ordination space, whereas the communities after crude oil treatment scattered away from them. Moreover, the oiled samples clustered as two separate groups. One is composed of the samples from the south (TY and JQ) and the other is made up of soils from north parts (WN and WL) along the CRCOP pipeline (Fig. 5 left). The NMDs ordination based on the Bray-Curtis distance (Fig. 5, right) generally agree with the CA analysis, showing clear bacterial shifts of bacterial populations in response to oil treatment. The oiled samples in ordination appeared to shift away from the corresponding clean/intact samples which grouped in the left part. Like the CA chart, the north samples (WN and WL) clustered together, while the south samples (TY and JQ) grouped more closely to each other, although not as strong as in the CA ordination. Furthermore, the overall microbial community structures were distinct site by site, but samples after treatment were generally clustered by geographic locations.

## Discussion

In many environments, a variety of native microorganisms are capable of petroleum degradation and they will dominate community following contamination. From this simulation experiment, treatment resulted in clear shift of bacterial communities in both the deep active layer and permafrost soils. The microbial community after contamination becomes dominated by those taxa capable of metabolizing or tolerant to complex hydrocarbons from the taxa of *Proteobacteria*, *Actinobacteria* and *Firmicutes*. The relative dominance of these bacteria varied between samples. Particularly, the group *Proteobacteria* dominated most clean and dirty soils, and this pattern was exaggerated following disturbance. The group of *Proteobacteria* typically dominates the microbial assemblage in hydrocarbon-contaminated soils, including alpine and polar soils, especially in the early stages of hydrocarbon degradation [22]. A pyrosequencing survey on oil sands tailing ponds indicated the taxa of *Proteobacteria*, *Firmicutes*, *Actinobacteria*, *Chloroflexi*, and *Bacteroidetes* were involved in anaerobic hydrocarbon degradation [23]. Enrichment of bacteria from the phyla *Chloroflexi*, *Firmicutes, Proteobacteria* was also found from crude oil degrading microcosms soil [24].

Among *Proteobacteria*, members of *Sphingomonadaceae* (esp. *Novosphingobium* and *Sphingobium*) drastically dominated in oiled samples, especially Walagan and Walagan North. *Sphingomonadaceae*, also known as *Sphingomonads* includes a versatile group which can effectively degrade a wide spectrum of hydrocarbon pollutants, especially aromatic petroleum compounds in template, polar soils or marine sediments [25]. *Sphingomonads* are more adapted to oligotrophic environments and are thus more competitive at low nutrient availability [26]. For example, an isolate from Antarctic soils (Ant 17) is able to degrade the aromatic compounds at 1°C without nutrient amendment [27]. In high Arctic soils, members of the *Sphingomonadaceae* were found to actively degrade diesel hydrocarbons [28]. Furthermore, some species from this family can alter membrane fluidity when hydrocarbon binds to cell membranes, permitting them to withstand contaminants in a relatively broad range of temperature [27]. Genetically, *Sphingomonad* has evolved a highly variable plasmid or chromosome-plasmid encoding numerous oxygenases and glycoside hydrolases which are involved in degrading numerous recalcitrant aromatic compounds. This feature provides a genetic basis for this lineage to perform competitively in ecology, metabolic versatility, and environmental adaptations [29].

Abundance of *Caulobacter* within *α-proteobacteria*, especially in Walagan samples was also enhanced after treatment. They had been found in cold Canadian arctic soils contaminated by diesel [28], [30]. Isolates of *Caulobacter* were also obtained from eight geographically distinct soils contaminated with crude oil [31]. Recently, a metagenomic approach showed that *Caulobacter* played critical roles in degrading alkane hydrocarbons [30]. Besides, *Caulobacter* species are able to break down PAHs [32]. For example, strain K31 adapted to a low-oxygen groundwater habitat was found to degrade chlorophenol in cold groundwater [33]. In particular, *Caulobacter* exists in ubiquitous nutrient-poor (“oligotrophic”) habitats, some species are even resistant to freezing [34]. Moreover, *Caulobacter* cells can produces adhesive stalks and attach themselves together that enhance nutrient uptake and biofilm formation [35]. Additionally, *Caulobacter* species are resistant to heavy metals [36], [37]. From the genetic perspective, the *Caulobacter* genome contains multiple clusters of genes encoding proteins essential for survival in a nutrient poor habitat, providing the organism with the ability to respond to a wide range of environmental fluctuations [32]. Therefore, *Caulobacter* should be exploitable for bioremediation applications at low nutrient availability or low temperature.

The phylotype of *Phenylobacterium* is especially prevalent in Tayuan and Jiagedaqi samples. Many strains in this genus can well utilize compounds with a phenyl-moiety, including synthetic substances such as chloridazon and antipyrin or analogues [38], [39]. Particularly, *Phenylobacterium* could break down recalcitrant herbicide like PyraminR with the participation of plasmids [40]. The type strain of *Phenylobacterium* holds the meta-cleaving enzyme decomposing complicated polycyclic compounds [41], [42]. *Phenylobacterium* together with some other oil degrading bacteria has been developed as a major component of bio-dispersion to remove hydrocarbon oil from marine environment [43]. Recently, species from this genus was formerly found in water-flooded petroleum reservoirs in the Dagang Oilfield, China [44]. Lately, abundant *Phenylobacterium* has been detected by 454 pyrosequencing in subsurface injection water samples in Algerian Oilfields [45]. More recently, *Phenylobacterium* members were identified to be active in utilizing a variety of motor fuels according to a field survey by 454 pyrosequencing [46]. However, their potential of degrading hydrocarbons remains less investigated. Nevertheless, the occurrence of *Phenylobacterium* in tested soil at least suggested its resistance to crude oil compounds, their degradation to petroleum hydrocarbons will be of interest in upcoming studies.

In addition to the microbes discussed above, some other taxa were enhanced in the oily samples but with limited abundances. *Arthrobacter* from *Actinobacteria* is very important in Tayuan sample. Members of this genus are usually found in hydrocarbon-contaminated environments. Many strains can utilize a wide range of halogenated aromatic compounds [47], [48], as well as homocyclic compounds and N-heterocycles [49]. Moreover, this genus has special resistance to both starvation and desiccation [50]. Some isolates from permafrost displayed strong resistance to repeated freezing-thawing processes [51]. These cold-tolerant *Arthrobacter* strains showed resistance to toxic hydrocarbons in cold environments [52]. The Antarctic degrader isolate could produce biosurfactant over a wide range of temperatures and pH values [53]. The biosurfactant can induce the alteration of cell membrane fluidity in case of contamination. Genetically, *Arthrobacter* comprises large plasmids related to degradation, which inherited through horizontal gene transfer [54]. Owing to these physiological and genetic features, *Arthrobacter* possibly take advantages to effectively breakdown persistent pollutants in cold environments.

Apart from *Arthrobacter*, *Acidovorax* became more abundant in the contaminated soils, especially for the Walagan samples. This taxon has been previously found to prevail in PAHs-contaminated soils [55] and have been involved in degrading PAHs [56], [57], particularly phenanthrene [57]–[59]. Experiment showed that *Acidovorax* substantially contributed to the dominant clones in soils contaminated by mineral oil over 60 years [60]. In cold arctic soils *Acidovorax* was also characterized as one of the few predominant bacterial types under aerobic or nitrate-reducing conditions at different temperatures [56]. Isotopic experiments implied that *Acidovorax* produced the most frequently encountered DNA fractions [59]. Particularly, an uncultured *Acidovorax* species was observed to still assimilate naphthalene at high concentrations that *Pseudomonas* isolates did not [61]. Concerning the abundant existence of *Acidovorax* in oil-amended samples, its roles in degrading oil aromatic hydrocarbons can be expected. More knowledge of *Acidovorax* under crude oil disturbance will be necessary for the *in-situ* bioremediation management.

In addition, crude oil also favored some other groups such as *Paenibacillus*, *Azovibrio*, *Cupriavidus*, *Pseudolabrys* and *Rhodococcus*. *Rhodococcus* is recognized as a key alkane degrader in contaminated polar soils [11]. But its abundance in this study is less than 5% except for Tayuan samples, possibly indicating the heavy crude oil was still eco-toxic for them. Concerning *Paenibacillus*, which is frequently detected in contaminated environments [62], some species within *Paenibacillus* are able to degrade PAHs [63], [64]. As endospore-forming bacteria, *Paenibacillus* can develop complex colonies by self-organization and cooperative behavior [62], [65]. However, their degradation capacity in the studied environments remains less explored. *Azovibrio* is capable of nitrogen fixation and fermentation [66]. Addition nitrogen to hydrocarbon-contaminated soils frequently favors growth of degraders when carbon is in abundance [22]. Therefore, *Azovibrio* might indirectly boost the bioremediation through its nitrogen metabolism. Coincidently, *Cupriavidus* from the family of *Burkholderiaceae* can also enhance biodegradation of soil pollutants by its nitrogen fixation [67].

From the ordination analysis, the clean samples are relatively more similar in relation to the oily samples. Even the clean sample also showed site-specific distribution in the ordination space. Furthermore, the microorganisms in the deep active layer and upper permafrost exhibited a certain consistency for each site. Greer *et al*. concluded that the initial microbial community structure in a soil is a function of the geographical location, the properties of the soil and the environmental conditions [22]. The addition of levels of contamination influenced the predominant microbial populations. Due to the selective pressure of crude oil exerted, the taxa those can degrade or resist crude oil components can gradually outnumbered in the communities. The communities will be gradually re-organized towards a new assemblage of hydrocarbon degraders. However, the crude oil disturbance did not result in highly similar bacterial profiles, rather, the community shifted, with site-to-site variations. Similar result has also been addressed previously [68], [69]. In this case, the resultant bacterial structure is more dependent on the geographical location than the levels of contamination, each plots contains site-specific predominance of degraders.

To sum up, this experiment revealed existence of the potential degraders within bacterial profiles, and clear shifts of bacterial community structure after oil amendment. The oiled samples were dominated by bacterial taxa of *Sphingomonadaceae* (mainly *Novosphingobium* and *Sphingomonas*), *Caulobacteraceae* (esp. *Caulobacter* and *Phenylobacterium*) and *Alicyclobacillaceae* (esp. *Alicyclobacillus*). Most survivors are either tolerant to or capable of degrading crude oil compounds, especially PAHs. However, the result should be slightly weakened by the insufficient sequencing depth and reflect the microbial response to conditions which are better than the *in-situ* condition. Even though the degraders are well adapted to the cold and sometimes nutrient-poor conditions, some conditions still need to be modified in order to optimize the microbial potential for degradation. Additional field studies with simulated contamination affecting the deep subsurface soils will definitely provide more understanding of microbial response to crude oil contamination. Notwithstanding its preliminary character, this study clearly indicates that both the deep active layer and upper permafrost contain a variety of indigenous hydrocarbon-degrading bacteria which can be potential *in-situ* agents of bioremediation if condition permitting.

## Supporting Information

Figure S1 **Observed OTU richness and estimated Chao1 and ACE indices for 16S rDNA libraries at 95% identity before and after crude oil treatment.**
(EPS)

Figure S2 **Rarefaction curve at cutoff of 0.03.**
(EPS)

Table S1 **Summary of the sampling sites in this experiment.**
(DOCX)
