# Supplementary material for: Crude Oil Treatment Leads to Shift of Bacterial Communities in Soils from the Deep Active Layer and Upper Permafrost along the China-Russia Crude Oil Pipeline Route
Source: PLoS One. 2014 May 2;9(5):e96552. doi: 10.1371/journal.pone.0096552 (PMC4008593; doi:10.1371/journal.pone.0096552)
Supplement: Table S1 — Summary of the sampling sites in this experiment. (DOCX) [file pone.0096552.s003.docx]

**Table S1**. Summary of the sampling sites in this experiment

| Sites | Coordinates | Permafrost table (cm) | Sampling layer | Depth (cm) | Soil type |
| --- | --- | --- | --- | --- | --- |
| Walagan North  (WN) | 52°43' N, 124°30' E | 80 | Bottom active layer | 70-80 | Peat with grey clay |
|  |  |  | Upper permafrost | 130-140 | Brownish clay with gravels |
| Walagan  (WL) | 52°26' N, 124°40' E | 80 | Bottom active layer | 70-80 | Peat with gravels |
|  |  |  | Upper permafrost | 150-160 | Gravels with brown clay |
| Tayuan  (TY) | 51°27' N, 124°15' E | 90 | Bottom active layer | 80-90 | Peaty clay |
|  |  |  | Upper permafrost | 120-130 | Grey clay with gravels |
| Jiagedaqi  (JQ) | 50°41' N, 124°17' E | 80 | Bottom active layer | 70-80 | Peaty clay |
|  |  |  | Upper permafrost | 140-150 | Grey clay with gravels |
